# Forest Management Type Influences Diversity and Community Composition of Soil Fungi across Temperate Forest Ecosystems

**DOI:** 10.3389/fmicb.2015.01300

**Published:** 2015-11-24

**Authors:** Kezia Goldmann, Ingo Schöning, François Buscot, Tesfaye Wubet

**Affiliations:** ^1^Department of Soil Ecology, Helmholtz Centre for Environmental Research-UFZHalle, Germany; ^2^Department of Biology II, University of LeipzigLeipzig, Germany; ^3^Max Planck Institute for BiogeochemistryJena, Germany; ^4^German Centre for Integrative Biodiversity Research (iDiv) Halle-Jena-LeipzigLeipzig, Germany

**Keywords:** soil fungal community, ectomycorrhizal fungi, temperate forest, forest management type, 454 pyrosequencing, rDNA

## Abstract

Fungal communities have been shown to be highly sensitive toward shifts in plant diversity and species composition in forest ecosystems. However, little is known about the impact of forest management on fungal diversity and community composition of geographically separated sites. This study examined the effects of four different forest management types on soil fungal communities. These forest management types include age class forests of young managed beech (*Fagus sylvatica* L.), with beech stands age of approximately 30 years, age class beech stands with an age of approximately 70 years, unmanaged beech stands, and coniferous stands dominated by either pine (*Pinus sylvestris* L.) or spruce (*Picea abies* Karst.) which are located in three study sites across Germany. Soil were sampled from 48 study plots and we employed fungal ITS rDNA pyrotag sequencing to assess the soil fungal diversity and community structure. We found that forest management type significantly affects the Shannon diversity of soil fungi and a significant interaction effect of study site and forest management on the fungal operational taxonomic units richness. Consequently distinct fungal communities were detected in the three study sites and within the four forest management types, which were mainly related to the main tree species. Further analysis of the contribution of soil properties revealed that C/N ratio being the most important factor in all the three study sites whereas soil pH was significantly related to the fungal community in two study sites. Functional assignment of the fungal communities indicated that 38% of the observed communities were Ectomycorrhizal fungi (ECM) and their distribution is significantly influenced by the forest management. Soil pH and C/N ratio were found to be the main drivers of the ECM fungal community composition. Additional fungal community similarity analysis revealed the presence of study site and management type specific ECM genera. This study extends our knowledge on the impact of forest management type on general and ectomycorrhizal fungal diversity and community structure in temperate forests. High plasticity across management types but also study site specific spatial distribution revealed new insights in the ECM fungal distribution patterns.

## Introduction

Soils, which are habitats for a wide range of microorganisms including fungi, are known to provide many ecosystem functions (Deacon, 2009). Saprotrophic and ectomycorrhizal (ECM) fungi play especially important roles in decomposition and nutrient cycling (Cairney and Meharg, 2002). Saprotrophic fungi take major part in decay processes (Hobbie et al., 1999), whereas ECM fungi are both decomposers and mutualists with plant roots and can constitute up to 80% of the fungal biomass in forest soils (Nehls, 2008). Soil fungal communities are shaped by a number of biotic and abiotic factors. Because of their close associations with plants, fungi show particularly high sensitivity to shifts in vegetation (Lauber et al., 2008). Trees, as the main plants in forest ecosystems, affect soil fungi passively by shading the ground and by regulating soil temperature and moisture as well as by influencing understory vegetation (Gömöryová et al., 2013). Differences in root architecture and exudation patterns (Aleklett and Hart, 2013; Bakker et al., 2014) are also known to impact soil properties such as nutrient concentrations (Jones et al., 2004) and pH (Hartmann et al., 2008), which directly affect the soil fungal community composition.

Forest management, i.e., any anthropogenic actions linked to timber production, can change the original composition of a forest through the removal and/or replacement of tree species, and by altering the age class structure, exporting biomass and modifying the proportion of dead wood (Paillet et al., 2010). Such changes initiate cascades of consequences that lead to modifications of abiotic and biotic factors both above- and below-ground (Paillet et al., 2010). In the natural state, Central Europe would be a deciduous forest (Bengtsson et al., 2000). However, the native vegetation in this area has been altered through management practices (Vanbergen et al., 2005). Currently less than 3% of the area of Central European forests is natural (FAO, 2010). For instance, the introduction of non-native coniferous species like Norway spruce (*Picea abies* Karst.) and Scots pine (*Pinus sylvestris* L.) represent one of the major changes in German forest within the last century (BMELV, 2011). The tree monocultures or human-induced fire events are known worldwide to change understory vegetation there by altering forest structures (Wingfield et al., 2001; Royo and Carson, 2006; Grant et al., 2007). In response to management practices, new types of microhabitats emerged which tend to result in a shift in the soil fungal and microbial communities. However, little is known about the effect of forest management regimes on soil fungal communities in Central European forests (Lang and Polle, 2011; Teste et al., 2012; Urbanová et al., 2015).

The German Biodiversity Exploratories, a long-term research platform located at three study sites in Germany, were implemented to assess the impact of forest management (with a focus on timber production) on the biodiversity and functions of forest ecosystems (Fischer et al., 2010). In this frame, previous surveys in the Biodiversity Exploratories investigating the effects of forest management on biodiversity were focusing on invertebrates in soil (Ferlian and Scheu, 2014; Klarner et al., 2014) or litter (Lange et al., 2011; Ott et al., 2014). At the microbial scale these effects of management were studied on soil bacterial communities (Nacke et al., 2011), wood-inhabiting bacteria (Hoppe et al., 2015), and wood-inhabiting fungi (Purahong et al., 2014) as well as soil fungi (Wubet et al., 2012) and yeasts (Yurkov et al., 2011) in beech-dominated forests. However, soil fungal communities under different forest management types within the Exploratories have not been studied so far.

To analyze the impact of four different forest management types within the three Exploratories (study sites; Fischer et al., 2010; Solly et al., 2014) on soil fungal diversity and community composition, we used the fungal ITS rDNA pyrotag sequencing approach. The main objectives of this study were: (i) to evaluate the impacts of study site and soil environmental conditions on fungal diversity and community composition, (ii) to assess the influence of forest management types on fungal diversity and community structure, and (iii) identify the effects of study site and forest management type on the relative distribution of ECM fungal taxa. We hypothesized that; (1) In accordance with the principle that ‘everything is everywhere but the environment selects’ (Bass Becking, 1934), different sites and management types will exhibit different levels of diversity and community composition of general and ECM fungi. (2) Forest management, changes in the main tree species and soil properties are important factors shaping the fungal community structure.

## Materials and Methods

### Study Sites

The study was performed as part of the Biodiversity Exploratories project, which uses three geographically separated study sites (Fischer et al., 2010). These sites are located in the south west (Swabian Alb), the centre (Hainich-Dün) and the north east (Schorfheide-Chorin) of Germany. Besides the variation in their topogeography they also differ with respect to their geology and climate (**Table 1**). We selected four forest management types according to Klarner et al. (2014). Briefly, the types are: age class forests of young managed beech (*Fagus sylvatica* L.), with beech stands age of approximately 30 years (B30); age class beech stands with an age of approximately 70 years (B70); unmanaged beech stands (unm B), and coniferous stands (Conif) consisting of pine (*Pinus sylvestris* L.) in Schorfheide-Chorin and spruce (*Picea abies* Karst.) in Swabian Alb and Hainich-Dün.

**Table 1:** Overview of the three study sites including: information about geography, climate, soil properties (Fischer et al., 2010; Solly et al., 2014); soil properties display the mean values (see section “Basic soil analyses”).

	Swabian Alb (Alb)	Hainich-Dün (Hai)	Schorfheide-Chorin (Sch)
**General information**			
Location	SW Germany	Central Germany	NE Germany
	N 48° 4′ E 9° 4′	N 51° 2′ E 10 ° 4′	N 53° 0′ E 13° 8′
**Climate**			
Annual mean temperature [°C]	6–7	6.5–8	8–8.5
Annual mean precipitation [mm]	700–1000	500–800	500–600
**Soil properties**			
Main soil type	Cambisol	Luvisol	Cambisol
Mean pH	5.07	4.99	3.39
Mean C_tot_[g/kg soil]	57.83	38.22	22.50
Mean N_tot_[g/kg soil]	4.27	2.82	1.22
Mean C/N ratio	13.45	13.42	18.54

### Soil Sample Collection

Soil samples were collected from four replicate experimental plots for each of the four forest management types in early May 2011 in parallel soil sampling campaigns conducted in all three study sites. On each of these 48 experimental plots (100 m × 100 m; Supplementary Table S1), 14 soil cores with a diameter of 5 cm were taken along two transects of 40 m length from north to south and from west to east at 1, 7, 13, 19, 31, and 37 m each. Organic layers were removed before taking the soil cores. For our study the upper 10 cm of the 14 soil cores from each plot were mixed. The composite samples were sieved at a mesh size of 2 mm. From the pooled soil samples, aliquots of 50 g were stored at -80°C for further molecular analysis. Additionally, a 500 g aliquot of soil was dried at 40°C for basic soil analyses.

### Basic Soil Analyses

All soil analyses were performed with air dried <2 mm samples. The pH was determined in duplicate, using a glass electrode in the supernatant of soil suspensions prepared using 1:2.5 mixtures of soil and 0.01 M CaCl_2_. Soil samples were ground to a size of 100 μm and analyzed for total carbon (C) and nitrogen (N) by dry combustion with a CN analyzer “Vario Max” (Elementar Analysensysteme GmbH, Hanau, Germany). After removal of organic C by combustion of samples for 16 h at 450°C, inorganic C was determined using the same method. Organic C concentrations were then calculated from the differences between total and inorganic C concentrations (Solly et al., 2014).

### DNA Extraction, Amplicon Library Preparation and Pyrosequencing

Microbial genomic DNA was extracted from two independent 0.5 g frozen subsamples of each soil sample collected during the sampling campaigns using a MO BIO Power Soil DNA isolation kit (MO BIO Laboratories, Carlsbad, CA, USA) following the manufacturer’s protocol. The two soil DNA extracts from each sample were pooled and DNA concentrations were quantified using a NanoDrop UV-Vis spectrophotometer (Peqlab Biotechnologie GmbH, Erlangen, Germany). The fungal ITS rDNA barcode region was amplified using custom ITS1F primers (Gardes and Bruns, 1993) containing Roche 454 pyrosequencing adaptor B and the universal primer ITS4 (White et al., 1990) containing Roche 454 pyrosequencing adaptor A and a sample-specific multiplex identifier (MID). The PCR reactions were performed as described previously (Wubet et al., 2012) in a total volume of 50 μl reaction mix containing 1 μl DNA template (7–15 ng), 25 μl Go Taq Green Master mix (Promega, Mannheim, Germany) and 1 μl of a solution containing 25 pmol of each of the ITS region-specific primers.

All samples were amplified in triplicate and purified using a Qiagen gel extraction kit (Qiagen, Hilden, Germany); DNA concentrations were then measured using a fluorescence spectrophotometer (Cary Eclipse, Agilent Technologies, Waldbronn, Germany) and the samples were pooled to give equimolar representation of each. Unidirectional pyrosequencing from the ITS4 end of the amplicons was performed using a 454 Titanium amplicon sequencing kit and the Roche GS-FLX + 454 pyrosequencer (Roche, Mannheim, Germany) at the Department of Soil Ecology, Helmholtz Centre of Environmental Research (UFZ, Halle, Germany). The raw ITS rDNA sequences were deposited in the National Center for Biotechnology Information (NCBI) Sequence Read Archive (SRA) under study accession number SRP049544.

### Bioinformatic Analyses and Ecological Grouping

Quality filtering and analysis of the 454 ITS sequences was performed in a sequential analysis using mainly MOTHUR (Schloss et al., 2009). In the initial filtering step, sequences with ambiguous bases, homo-polymers and primer differences of more than eight bases were removed. Simultaneously all primer and barcode sequences were discarded. At the same time, sequence reads with a quality score lower than 20 and a read length of less than 300 bp were removed, using the keepfirst 300 bp command and thereby chopping at least 50 bp of the sequence end to remove sequencing noise. This resulted in a sequence read fragment of 300 bp length covering the ITS2 region. All samples were normalized to the smallest sample size (2040 reads per sample) by random removal using the subsample command as implemented in MOTHUR. Sequences were checked for chimeric sequences using the UCHIME algorithm (Edgar et al., 2011) implemented in MOTHUR. The remaining, non-chimeric, sequences were clustered into operational taxonomic units (OTUs) using cd-hit-est (Li and Godzik, 2006) at a threshold of 97% pairwise identity. Taxonomic assignment of the representative sequences for the OTUs was done with the classify.seq command of MOTHUR applied to the UNITE fungal ITS reference database version 6 (Kõljalg et al., 2013). To improve the taxonomical resolution, those OTUs that had been identified only down to the family level were then subjected to a BLASTn search (e.g., Johnson et al., 2008) against the NCBI GenBank database (Benson et al., 2015). Finally, the fungal OTUs that had been assigned at the genus level were put into ecological groups on the basis of information from literature, in order to link taxonomic information to potential functions.

### Statistical Analyses

Statistical analyses were performed using the softwares R version 3.1.1 (R Development Core Team, 2008) and PAST version 2.17b (Hammer et al., 2001).

In order to define the data matrix for our statistical analyses addressing our objectives and verify our hypothesis, we first tested the effect of removing rare fungal taxa on community composition. To assess the influence of rare fungal OTUs (OTUs represented by ≤3 reads), we calculated the non-metric multidimensional scaling (NMDS) ordination with 20 random starts from the dataset both with all OTUs and with only the abundant fungal OTUs (OTUs represented by >3 reads). The congruence between the two ordination sets was tested by Procrustes correlation analysis using the protest function (Peres-Neto et al., 2006) of the R package vegan (Oksanen et al., 2015) with 999 permutations. We found that fungal community composition was not significantly affected by the presence or absence of rare fungal OTUs (Procrustes correlation coefficient = 0.9985; *p* < 0.001, suggesting nearly identical ordination). We also tested the need for re-normalization of the abundant fungal OTU data matrix and compared the congruence of the NMDS plots based on the dominant fungal OTU data matrix and on a re-normalized abundant OTU data matrix using Procrustes correlation analysis. We found that this normalization step did not affect the fungal community composition (Procrustes correlation coefficient = 0.9995; *p* < 0.001). Hence, all subsequent analyses were performed using the relative abundance fungal community matrix excluding singletons, doubletons and tripletons.

Fungal OTU diversity was assessed by calculating the Shannon-Wiener diversity index (Shannon, 1948) using the diversity function in vegan (Oksanen et al., 2015). Effects of study site and forest management type on general and ectomycorrhizal fungal OTU richness and Shannon diversity as well as soil chemical properties were tested using two-way analysis of variances (ANOVA) followed by a Tukey *post hoc* test. Correlation analyses were performed to test the association between general and ectomycorrhizal fungal OTU richness, respectively, Shannon diversity and soil chemical properties. Whether the correlations were positive or negative was revealed by linear modeling. To assess similarities in the general and ectomycorrhizal fungal community structure among the three study sites, we performed two-way non-parametric multivariate analysis of variances (NPMANOVA) using PAST (Hammer et al., 2001). Relationships between fungal communities and forest management type within the three study sites were visualized using NMDS on the basis of a Bray–Curtis distance matrix and 30 random starts using the metaMDS and ordihull functions of the vegan package (Oksanen et al., 2015). The function envfit was used to test the goodness of fit of environmental parameters on the fungal community NMDS ordination plot based on 999 random permutations. Significant correlations were plotted as vectors.

The relative contributions made by the ecological group of ectomycorrhizal fungal taxa (ECM) to community composition under the different forest management types and different study sites was assessed by means of similarity percentage analysis (SIMPER) based on the relative abundance of the fungal OTUs using PAST (Hammer et al., 2001). This method compares average relative abundance and examines the contribution of each ECM genus to the observed overall dissimilarities between groups or similarities within a given group (Clarke and Warwick, 2001; Gosling et al., 2013). The percentage contributions of the ECM genera, which add up to a total of 90% of the observed dissimilarities between the four different management types, across the three study regions were additionally visualized on heatmaps plotted in R using the package ggplot2 (Wickham, 2009). Significance of differences in the relative abundance of ECM in the respective forest management types were tested by a one-way ANOVA, comparing the mean relative abundance of each genus. To test individual effect of soil properties on ECM genera within the respective forest management types, we performed correlation analysis between the mean abundances with soil pH and C/N.

## Results

### Sequence Quality Control and Characterization of Soil Fungi

From the 48 soil samples, consisting of four replicates of four different forest management types collected at each of three study sites, a total of 201,381 reads were obtained. Subsequent sequence quality filtering and normalization resulted in 97,920 sequences representing 2040 reads per sample. Further removal of a total of 3113 potential chimeric and non-fungal sequences resulted in 5333 fungal OTUs including 3710 rare OTUs. As described in detail in the section “Material and Methods”, neither removal of rare taxa nor re-normalization of the dominant data matrix had a significant effect on the composition of the fungal community (the number of sequences in each dataset is given in Supplementary Table S2). Consequently only the 1623 abundant fungal OTUs were used for subsequent analysis.

Taxonomic assignment showed that Basidiomycota represent the most diverse fungal phylum, with 858 OTUs (53% of the total), followed by Ascomycota (405 OTUs, 25%), Zygomycota (68 OTUs, 4.2%), Chytridiomycota (10 OTUs, 0.6%), Glomeromycota (10 OTUs, 0.6%) and basal fungi (only assigned down to kingdom level, 272 OTUs; Supplementary Figure S1A). In addition, 61.4% of the fungal OTUs (996 OTUs) were taxonomically classified down to the genus level. Subsequently these taxa were assigned to groups reflecting their ecological function, including: 38.1% (618 OTUs) ectomycorrhizal and 18.9% (306 OTUs) non-mutualistic fungi (saprotrophs, parasites, and pathogens as well as endophytic) fungal genera. The remaining OTUs belong to either potential mutualists (1.5%, 25 OTUs), other symbionts such as arbuscular and ericoid mycorrhiza or lichens (1.2%, 19 OTUs). For the remaining 655 OTUs (40.36%) their ecological functions are yet unknown. The proportion of the ecological functions of the fungal OTUs is displayed in Supplementary Figure S1B.

### Effects of Study Site and Forest Management Type on Fungal OTU Richness, Shannon Diversity and Soil Properties

Analysis of the impact of study site and forest management type on general fungal OTU richness and Shannon diversity using a two-way ANOVA showed that study site had no impact on fungal diversity and fungal richness. In contrast, Shannon diversity was significantly influenced by forest management type. Furthermore, Shannon diversity and OTU richness were affected by the interaction of study site and forest management type (**Table 2**). A Tukey *post hoc* test indicated that the lowest diversity was found in the unmanaged beech forests of the Swabian Alb which was significantly smaller than in young beech forests of the same study site, unmanaged beech stands in Hainich-Dün, and coniferous forests in Schorfheide-Chorin (Supplementary Figure S2). However, we found no significant impact of the forest management type within the study sites on fungal OTU richness. In contrast to total fungal OTU richness, two-way ANOVA showed the effect of study site and forest management type on ECM fungi, the major ecological group in this study (**Table 2**). The unmanaged beech and coniferous forest management types in Schorfheide-Chorin showed significantly lower OTU richness than in old and unmanaged beech forests in the two study sites (Swabian Alb and Hainich-Dün, Supplementary Figure S3).

**Table 2:** The impact of study site and forest management type on fungal operational taxonomic units (OTU) richness, Shannon diversity of all genereal and ECM fungi, carbon and nitrogen contens, C/N ratio and pH based on a two-way ANOVA.

		Study site	Forest management type	Study site:forest management type
OTU richness	*p*	0.447	0.202	<0.05	
	*F*	0.823	1.619	2.513	
Shannon diversity	*p*	0.064	<0.05	<0.05	
	*F*	2.971	3.314	2.774	
ECM OTU richness	*p*	<0.001	<0.05	0.509	


	*F*	16.124	5.575	0.892	
ECM Shannon diversity	*p*	<0.05	0.427	0.392	


	*F*	7.634	0.951	1.1081	
*C*_tot_	*p*	<0.001	0.189	0.238	
	*F*	4.568	1.676	1.405	
*C*_inorg_	*p*	<0.05	0.298	<0.05	
	*F*	6.036	1.274	2.451	
*C*_org_	*p*	<0.001	0.211	0.285	
	*F*	44.305	1.582	1.294	
*N*_tot_	*p*	<0.001	0.627	0.476	
	*F*	53.161	0.58	0.944	
C/N ratio	*p*	<0.001	<0.001	0.682	
	*F*	81.954	7.021	0.66	
pH	*p*	<0.001	0.652	<0.05	
	*F*	36.251	0.549	4.064	

*F statistics – mean of the within group variances and *p* – significance value (in bold are *p* < 0.05)*.

Further two-way ANOVA to assess the effects of study site and forest management type on the soil properties revealed that study site had a significant impact on all six tested soil properties, whereas forest management type affected only C/N ratio (**Table 2**). For instance, pH was in general lower in Schorfheide-Chorin. Particularly, the soil in old beech forest in this study site was more acidic than in young and unmanaged beech forest in Swabian Alb and coniferous forest in Hainich-Dün (Supplementary Figure S4). In contrast, in Schorfheide-Chorin all observed forest management types showed a significant higher C/N ratio. An exception are the unmanaged beech forests in this study site because they were not significantly different from the coniferous forests in the other two study sites (Swabian Alb and Hainich-Dün, Supplementary Figure S4).

Soil properties did not correlate significantly with either general fungal OTU richness or with the Shannon diversity. Ectomycorrhizal fungal OTU richness and Shannon diversity, however, showed positive correlations with pH and negative correlations with C/N ratio (Supplementary Table S3).

### Fungal Community Similarity and Factors Correlating with Community Composition

**FIGURE 1:**
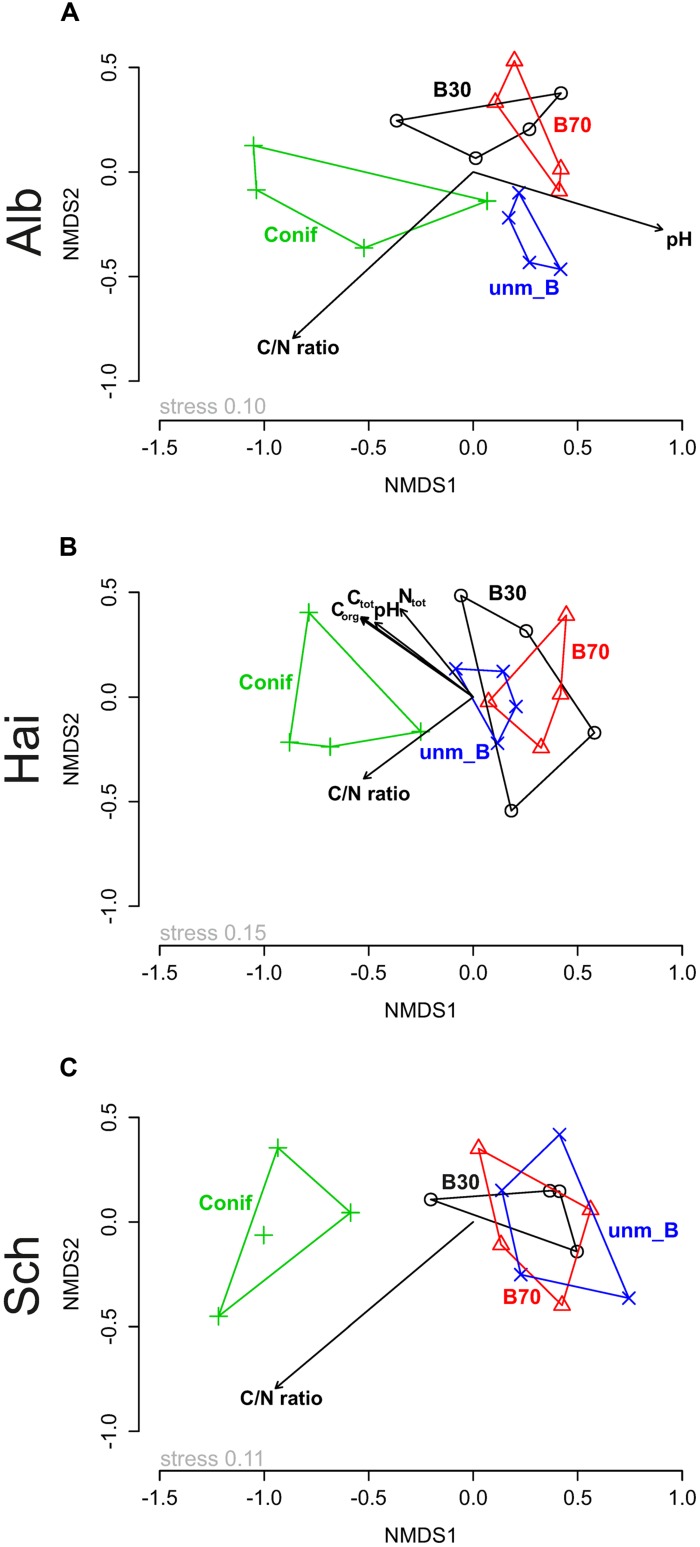
**Non-metric multidimensional scaling (NMDS) ordination displaying fungal community composition across the three study sites, **(A)** Swabian Alb (Alb), **(B)** Hainich-Dün (Hai), and **(C)** Schorfheide-Chorin (Sch), in relation to forest management type (o B30–young beech forest, Δ B70–old beech forest, + Conif–coniferous forest, x unm_B–unmanaged beech forest) and soil properties (*C*_tot_–total carbon, *C*_org_–organic carbon, *N*_tot_–total nitrogen, C/N ratio–ratio of organic carbon to total nitrogen and pH).** Stress values represent percentage.

Comparisons of fungal community similarity among the three study sites using two-way NPMANOVA revealed differences in community composition under the different forest management types (*F* = 1.885, *p* < 0.05), study sites (*F* = 5.774, *p* < 0.05) and a significant interaction between the two factors (*F* = 1.481, *p* < 0.05). Consequently, we performed one-way NPMANOVA to assess the pairwise effect of both study sites and forest management (**Table 3**). The results indicated that the fungal community composition at Schorfheide-Chorin differed significantly from those in Swabian Alb and Hainich-Dün (*p* < 0.05; see **Table 3**) and that the community composition under conifer differs always from the communities under beech (*p* < 0.05, **Table 3**). Similarly, NMDS based ordination of the fungal community displayed the correlation of study site (*p* < 0.05; Supplementary Figure S5A) and forest management type (*p* < 0.05, Supplementary Figure S5B). Study site based NMDS ordination plot analysis also revealed the correlation with forest management type in the respective study sites (**Figure 1**). In addition, soil chemical properties also contributed significantly to the observed differences in fungal community composition among the three study sites (displayed as vectors in **Figures 1A–C** and summarized in Supplementary Table S4). Although C/N ratio showed a consistent correlation with the fungal community composition at all three study sites, the effect of pH was detected only at Swabian Alb and Hainich-Dün (**Figures 1A,B**).

**Table 3:** Community comparison among the three study sites and four forest management types.

	General fungi		Ectomycorrhizal fungi	
	*F*	*p*	*F*	*p*
**Study sites**				
Alb vs. Hai	1.628	0.051	1.534	0.057
Alb vs. Sch	7.819	**<0.05**	7.718	**<0.05**
Hai vs. Sch	6.77	**<0.05**	6.679	**<0.05**
**Forest management type**				
B30 vs. B70	0.7353	1	0.6293	1
B30 vs. unm_B	0.9926	1	0.86	1
B30 vs. Conif	1.785	**<0.05**	1.746	**<0.05**
B70 vs. unm_B	0.9357	1	0.9242	1
B70 vs. Conif	2.198	**<0.05**	2.2056	**<0.05**
Unm_B vs. Conif	2.179	**<0.05**	1.981	**<0.05**

*Pairwise comparison of community composition among the three study sites and four forest management types was done based on non-parametric multivariate analysis of variance (NPMANOVA; *n* = 16 for study sites and *n* = 12 for management types). Test statistics include *F* and *p-*values were based on 999 permutations (in bold are *p* < 0.05). Abbreviations: Swabian Alb – Alb, Hainich-Dün – Hai, Schorfheide-Chorin – Sch, young beech forest – B30, old beech forest – B70, unmanaged beech forest – unm_B and coniferous forest – Conif*.

The comparison of the similarity matrices of the whole fungal community with the one of the ECM revealed that the ordination was up to 88% identical (Procrustes correlation coefficient = 0.883, *p* < 0.05), suggesting that the community composition of ECM fungi followed the pattern of the whole fungal community. The ECM fungal communities in Schorfheide-Chorin differed significantly from those in Swabian Alb and Hainich-Dün (*p* < 0.05, Supplementary Figure S5C). A similar trend was found for the effects of forest management with distinct communities under conifers as compared to beech-dominated stands (*p* < 0.05; Supplementary Figure S5D). These findings were supported by a two-way NPMANOVA, which revealed the effect of study site (*F* = 5.602, *p* < 0.05), forest management type (*F* = 1.744, *p* < 0.05) and their interaction effect (*F* = 1.440, *p* < 0.05) on the ECM fungal community. Indeed, pairwise effects of study site and forest management type followed the same trends than for the general soil fungi (**Table 3**). The correlation with soil chemical properties was absent in Schorfheide-Chorin. However, in Swabian Alb pH and C/N ratio corresponded significantly to the ECM fungal community composition. In contrast, at Hainich-Dün all tested parameters except C/N ratio significantly affected the ECM community (Supplementary Table S5).

### Diversity and Community Composition of Ectomycorrhizal Fungal Taxa

**FIGURE 2:**
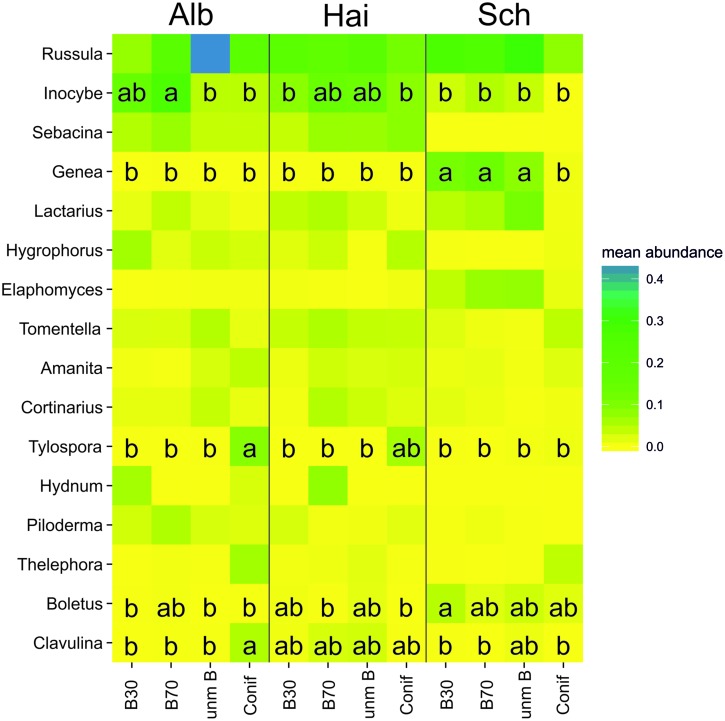
**Distribution of mean abundance of the most important ectomycorrhizal genera across the forest management types (young beech forest–B30, old beech forest–B70, coniferous forest–Conif, and unmanaged beech forest–unm B) at the study sites Alb - Swabian Alb, Hai - Hainich-Dün and Sch- Schorfheide-Chorin visualized on a heatmap.** Mean abundances based on SIMPER using Bray–Curtis distance, plotting ectomycorrhizal genera contributing to a cumulative percentage of 90%. Different letters within squares indicate significant differences (*p* < 0.05) based on Tukey *post hoc* pairwise comparison.

Analysis of percentage similarity (SIMPER) and heatmap-based visualization of the ECM community showed the relative importance of the different fungal genera across the three study sites in relation to the four forest management types (overall dissimilarity = 63.07%; **Figure 2**). We detected 16 ectomycorrhizal genera that contributed to a cumulative percentage of 90% of the overall variation at all three study sites. All the 16 ECM genera appeared in Swabian Alb and Hainich-Dün, while the genera *Sebacina* and *Hydnum* were completely absent in Schorfheide-Chorin. The relative abundance of the ECM fungal genera detected in this study also differed among the three study sites. Furthermore, as a result of forest management types, those ECM fungal genera that are of high abundance and/or importance under beech forest management types are less so in coniferous forests, e.g., *Genea* in Schorfheide-Chorin, and vice versa, e.g., *Tylospora* in Swabian Alb and Hainich-Dün. Correlation analysis of soil pH and C/N ratio with the ECM genera revealed very few significant relationships (Supplementary Table S6). Nonetheless, we found no clear pattern between ECM features like exploration type or hydrophobicity and soil pH or C/N ratio.

## Discussion

### Fungal OTU Characterization

In our study we found that 1263 OTUs (≈78% of all abundant OTUs) belonged to the Asco- or Basidiomycota. Although the phylum of the Ascomycota is the largest among the Fungi (James et al., 2006) we detected more basidiomycetous OTUs in the temperate forest in this study. Of the OTUs identified down to the genus level and classified by ecological function, 38% were ECM fungi. The fungal partners in most ECM symbioses are dominant members of the Basidiomycota followed by Ascomycota (Smith and Read, 2008). The importance of ECM fungi in temperate forest ecosystems may therefore explain the relatively high proportion of basidiomycetous OTUs in our dataset. The primer pairs used in this study have been widely used to amplify the whole fungal ITS rDNA region (Amend et al., 2010; Ihrmark et al., 2012; Wubet et al., 2012). Nevertheless, it is well know that these primer pairs tend to favor Asco- and Basidiomycota over other fungal taxa.

### Fungal Diversity Relations with Study Site and Forest Management

We found a significant interaction effect of study site and forest management type on both general fungal Shannon diversity and OTU richness (**Table 2**). This is mainly observed within the Swabian Alb young beech forests composed of more diverse fungi than the unmanaged beech forest. We assume that the recurrent disturbances that occur in managed stands (like B30 or Conif) could lead to higher fungal diversity, since ECM and other plant-associated fungi are known to be vulnerable to disturbance (Lazaruk et al., 2005; Gömöryová et al., 2013). In contrast, thinning improves the root growth of the remaining trees (Grant et al., 2007; Lin et al., 2011) and provides new habitats which could lead to an increase in fungal diversity. Thinning processes also alter light conditions, making it possible for other plants to fill the gaps (Brunet et al., 2010). However, plant diversity is not necessarily reflected in below-ground diversity (Gömöryová et al., 2013). We also found that unmanaged beech plots in Swabian Alb were less diverse than plots under highly managed stands (B30). Unmanaged beech forests include several features, such as decaying trees, different forms of deadwood, and uneven relief (mounds and pits; Bengtsson et al., 2000; Paillet et al., 2010), which lead to an expected greater fungal diversity within the soil environment. This could be only shown in Hainich-Dün where the Shannon diversity was among the highest in this forest management type. Recently Mölder et al. (2014) has reported a reduction of forest management leads to dominance of European beech and simultaneously to a decrease in understory vegetation. These authors suggested that dominance of one tree species could maintain an increase in fungal diversity (Paillet et al., 2010; Mölder et al., 2014), which is not thoroughly demonstrated by our results (Supplementary Figure S2). Moreover, in Schorfheide-Chorin the observed differences in the diversity and richness ECM fungal communities could be attributed to the soil properties. Soil pH was significantly and positively correlated to the observed OTU richness of ectomycorrhizal fungal while C/N was correlated significantly and negatively. This is in accordance with previous studies (Wubet et al., 2012; Tedersoo et al., 2014).

### Role of the Main Tree Species in Shaping the Fungal Community Composition

The results of NMDS analysis revealed significant variation in the composition of the fungal community among the three study sites and management types within the study sites (**Figure 1**). In the respective study sites, the general and ectomycorrhizal fungal community under coniferous forest differed significantly from those found in beech-dominated stands. Fungi, in particular mutualists and decomposers, show high sensitivity to changing vegetation (Lauber et al., 2008), implying the effect of tree species as the “most important ‘filter’ ” for shaping the fungal and general microbial community in root surrounding soils (Aleklett and Hart, 2013). This importance is due to various root traits. Deciduous tree species found in temperate forests have root architectures that are very different from those of the coniferous trees (specifically European beech, Norway spruce, and Scots pine) present in these forests (Kutschera and Lichtenegger, 2002). The development of shallow root systems, in comparison to deep rootedness, could explain differences in fungal community structures as seen in our results. Furthermore the presences of only one host species due to the plantation management practice (i.e., Norway spruce – *P. abies* Karst.), which provides only one kind of litter, could also lead to slight shifts in fungal communities. In general, our results indicated that fungal communities under beech management types did not differ significantly, suggesting no effect of the management types or age class among the beech forests.

Forest management types can influence the vertical stratification of forests. For example, regular timber harvests lead to more compact soil and affect the herb layer (Godefroid and Koedam, 2004). As a consequence, changes in the soil fungal community structure are to be expected. Soil pH, shaping soil fungal communities, can also affect patterns of herbal plants in forests (Brunet et al., 2010) and consequently the soil microbial community. Thus, the lower pH at Schorfheide-Chorin could additionally explain the distinctness of the fungal community as compared to those at the other two study sites. These results on the effect of soil pH are in line with a number of reports on the role of soil pH in shaping fungal communities in soil ecosystems (see review by Aleklett and Hart, 2013). Although pH depends mainly on soil type, texture, and the parental rock material, the plant community and its root deposits can also alter soil acidity (Jones et al., 2004; Lauber et al., 2008). Due to leaching and bioturbation processes, litter influences substrate availability in the topsoil (Kasel et al., 2008) and this may explain why we found a consistent effect of the C/N ratio on fungal community in all the three study sites (**Figure 1**).

### Relationship between the Main Tree Species and ECM Fungal Community

Fungal community similarity analysis revealed the presence of study site and management type specific ECM genera. Due to comparable soil properties (**Table 1**), all of the ECM fungal genera were shared between the Hainich-Dün and Swabian Alb study sites. In contrast, in Schorfheide-Chorin only 14 ECM genera were found as members of the ECM communities. Strong variations in the soil properties and organic matter substrates might account to the observed distinct ECM communities (Walker et al., 2014). The abundant ECM genera, *Russula*, *Inocybe*, and *Lactarius*, are known to be widely distributed ECM fungi (Kirk et al., 2008; Tedersoo et al., 2010). Although *Russula* has mainly been described as being distributed in coniferous forests (Geml et al., 2010; Tedersoo et al., 2010), we were able to show that this genus also occurs in beech dominated deciduous forests, particularly in the case of Schorfheide-Chorin, we found it to be abundant in beech-dominated forests. Consistent with the report of Twieg et al. (2007), who observed significant differences in fungal community composition between stands of different ages in both Douglas-fir and paper birch, we detected the trend of an increase in the genus *Russula* with increasing forest age in beech stands (**Figure 2**). In contrast to boreal forests, where the relative abundance of ECM DNA was higher in younger stands (Clemmensen et al., 2015), our study showed lower ECM abundance in young temperate beech forests.

In addition, we observed the presence of ECM genera that were specific to coniferous or to beech forest. For instance, in Swabian Alb and Hainich-Dün clear differences were found between the beech and coniferous stands, with the genus *Tylospora* being significantly more abundant in the coniferous stands. This corresponds to recent findings (Miyamoto et al., 2015) and is in line with previous reports of strong host species preference by ECM fungi (e.g., Ishida et al., 2007; Gao et al., 2013). However, *Lactarius*, another major genus of the family Russulaceae (Kirk et al., 2008) and one which has already been described in coniferous forest (Pande et al., 2004; Wang, 2007; Ilyas et al., 2013) was almost absent under the coniferous management types in our study. However, this genus was very abundant under all beech management types, particularly at Hainich-Dün and Schorfheide-Chorin. Our results also showed that taxa like *Cenococcum*, which are commonly found in morphotyping studies in temperate forests (Jany et al., 2003; Goicoechea et al., 2009), were not among the prominent ECM genera at our study sites. Previous studies were able to display relationships between ECM exploration strategies (Agerer, 2001) and nutrient uptake under different soil chemical properties (Suz et al., 2014; Clemmensen et al., 2015; Sterkenburg et al., 2015). Although our results revealed the effect of soil pH on the short-range exploration ECM types *Inocybe*, *Genea*, and *Hydrophorus* in our analysis the significant correlations appear very scattered (Supplementary Table S6B) and do not support the concept of replacements of explorations types with changing soil fertility (Sterkenburg et al., 2015).

## Conclusion

In general, this study was able to show that forest management types at more or less similar but geographically separated study sites induced a high level of distinctions in the composition of soil fungal communities in general and ECM fungi in particular. High aboveground heterogeneity, like in unmanaged beech forests, does not automatically result in high diversity of soil fungi (Gömöryová et al., 2013). More disturbed sites, such as young beech forests and highly managed coniferous stands, were found to be composed of diverse fungal communities. Dominant tree species in addition with soil properties are the main factors shaping fungal communities in temperate forests. Nevertheless, our analysis of the most important ECM fungi showed that the distribution pattern of certain taxa is more complicated and can only partly be explained by the effects of host tree species, soil pH or C/N ratio. Factors such as understory vegetation, climatic variation, rooting depths and root exudation profiles could also contribute in shaping the fungal communities and therefore should be considered in future studies. Temporal and spatial long-term monitoring of soil fungi in forest ecosystems also remains crucial to discover fungal diversity and ecosystem functioning.

## Conflict of Interest Statement

The authors declare that the research was conducted in the absence of any commercial or financial relationships that could be construed as a potential conflict of interest.

## References

[B1] Agerer R. (2001). Exploration types of ectomycorrhizae. *Mycorrhiza* 11 107–114. 10.1007/s005720100108

[B2] Aleklett K. Hart M. (2013). The root microbiota—a fingerprint in the soil? *Plant Soil* 370 671–686. 10.1007/s11104-013-1647-7

[B3] Amend A. S. Seifert K. A. Bruns T. D. (2010). Quantifying microbial communities with 454 pyrosequencing: does read abundance count? *Mol. Ecol.* 19 5555–5565. 10.1111/j.1365-294X.2010.04898.x 21050295

[B4] Bakker M. G. Schlatter D. C. Otto-Hanson L. Kinkel L. L. (2014). Diffuse symbioses: roles of plant–plant, plant–microbe and microbe–microbe interactions in structuring the soil microbiome. *Mol. Ecol.* 23 1571–1583. 10.1111/mec.12571 24148029

[B5] Bass Becking L. (1934). *Geobiologie of Inleiding tot de Milieukunde.* Hague: WP Van Stockum & Zoon.

[B6] Bengtsson J. Nilsson S. G. Franc A. Menozzi P. (2000). Biodiversity, disturbances, ecosystem function and management of European forests. *For. Ecol. Manage.* 132 39–50. 10.1016/S0378-1127(00)00378-9

[B7] Benson D. A. Clark K. Karsch-Mizrachi I. Lipman D. J. Ostell J. Sayers E. W. (2015). GenBank. *Nucleic Acids Res.* 43 D30 10.1093/nar/gku1216 PMC4383990 25414350

[B8] BMELV (2011). *German Forests - Nature and Economic Factor. Federal Ministry of Food, Agriculture and Consumer Protection.* Berlin: BMELV.

[B9] Brunet J. Fritz Ö Richnau G. (2010). Biodiversity in European beech forests–a review with recommendations for sustainable forest management. *Ecol. Bull.* 53 77–94.

[B10] Cairney J. W. G. Meharg A. A. (2002). Interactions between ectomycorrhizal fungi and soil saprotrophs: implications for decomposition of organic matter in soils and degradation of organic pollutants in the rhizosphere. *Can. J. Bot.* 80 803–809. 10.1139/b02-072

[B11] Clarke K. R. Warwick R. M. (2001). *Change in Marine Communities: An Approach to Statistical Analysis and Interpretation.* Plymouth Marine Laboratory: PRIMER-E.

[B12] Clemmensen K. E. Finlay R. D. Dahlberg A. Stenlid J. Wardle D. A. Lindahl B. D. (2015). Carbon sequestration is related to mycorrhizal fungal community shifts during long-term succession in boreal forests. *New Phytol.* 205 1525–1536. 10.1111/nph.13208 25494880

[B13] Deacon J. W. (2009). *Fungal Biology.* New York, NY: John Wiley & Sons.

[B14] Edgar R. C. Haas B. J. Clemente J. C. Quince C. Knight R. (2011). UCHIME improves sensitivity and speed of chimera detection. *Bioinformatics* 27 2194–2200. 10.1093/bioinformatics/btr381 21700674 PMC3150044

[B15] FAO (2010). *Global Forest Resources Assessment 2010.* Rome: FAO, 343.

[B16] Ferlian O. Scheu S. (2014). Shifts in trophic interactions with forest type in soil generalist predators as indicated by complementary analyses of fatty acids and stable isotopes. *Oikos* 123 1182–1191. 10.1111/j.1600-0706.2013.00848.x

[B17] Fischer M. Bossdorf O. Gockel S. Hänsel F. Hemp A. Hessenmöller D. (2010). Implementing large-scale and long-term functional biodiversity research: the biodiversity exploratories. *Basic Appl. Ecol.* 11 473–485. 10.1016/j.baae.2010.07.009

[B18] Gao C. Shi N.-N. Liu Y.-X. Peay K. G. Zheng Y. Ding Q. (2013). Host plant genus-level diversity is the best predictor of ectomycorrhizal fungal diversity in a Chinese subtropical forest. *Mol. Ecol.* 22 3403–3414. 10.1111/mec.12297 24624421

[B19] Gardes M. Bruns T. (1993). ITS primers with enhanced specificity for basidiomycetes – application to the identification of mycorrhizae and rusts. *Mol. Ecol.* 2 113–118. 10.1111/j.1365-294X.1993.tb00005.x 8180733

[B20] Geml J. Laursen G. A. Herriott I. C. Mcfarland J. M. Booth M. G. Lennon N. (2010). Phylogenetic and ecological analyses of soil and sporocarp DNA sequences reveal high diversity and strong habitat partitioning in the boreal ectomycorrhizal genus *Russula* (*Russulales*; *Basidiomycota*). *New Phytol.* 187 494–507. 10.1111/j.1469-8137.2010.03283.x 20487310

[B21] Godefroid S. Koedam N. (2004). Interspecific variation in soil compaction sensitivity among forest floor species. *Biol. Conserv.* 119 207–217. 10.1016/j.biocon.2003.11.009

[B22] Goicoechea N. Closa I. De Miguel A. M. (2009). Ectomycorrhizal communities within beech (*Fagus sylvatica* L.) forests that naturally regenerate from clear-cutting in northern Spain. *New For.* 38 157–175. 10.1007/s11056-009-9137-8

[B23] Gömöryová E. Ujházy K. Martinák M. Gömöry D. (2013). Soil microbial community response to variation in vegetation and abiotic environment in a temperate old-growth forest. *Appl. Soil Ecol.* 68 10–19. 10.1016/j.apsoil.2013.03.005

[B24] Gosling P. Mead A. Proctor M. Hammond J. P. Bending G. D. (2013). Contrasting arbuscular mycorrhizal communities colonizing different host plants show a similar response to a soil phosphorus concentration gradient. *New Phytol.* 198 546–556. 10.1111/nph.12169 23421495 PMC3798118

[B25] Grant C. D. Norman M. A. Smith M. A. (2007). Fire and silvicultural management of restored bauxite mines in Western Australia. *Restor. Ecol.* 15 S127–S136. 10.1111/j.1526-100X.2007.00300.x

[B26] Hammer, Ø Harper D. A. Ryan P. D. (2001). PAST: paleontological statistics software package for education and data analysis. *Palaeontol. Electron.* 4:9.

[B27] Hartmann A. Schmid M. Tuinen D. V. Berg G. (2008). Plant-driven selection of microbes. *Plant Soil* 321 235–257. 10.1007/s11104-008-9814-y

[B28] Hobbie E. A. Macko S. A. Shugart H. H. (1999). Insights into nitrogen and carbon dynamics of ectomycorrhizal and saprotrophic fungi from isotopic evidence. *Oecologia* 118 353–360. 10.1007/s004420050736 28307279

[B29] Hoppe B. Krüger D. Kahl T. Arnstadt T. Buscot F. Bauhus J. (2015). A pyrosequencing insight into sprawling bacterial diversity and community dynamics in decaying deadwood logs of *Fagus sylvatica* and *Picea abies*. *Sci. Rep.* 5:9456 10.1038/srep09456 PMC4389208 25851097

[B30] Ihrmark K. Bodeker I. T. Cruz-Martinez K. Friberg H. Kubartova A. Schenck J. (2012). New primers to amplify the fungal ITS2 region–evaluation by 454-sequencing of artificial and natural communities. *FEMS Microbiol. Ecol.* 82 666–677. 10.1111/j.1574-6941.2012.01437.x 22738186

[B31] Ilyas S. Razaq A. Khalid A. N. (2013). Molecular investigations to determine the ectomycorrhizal habit of *Lactarius sanguifluus* associated with coniferous and deciduous vegetation of Galyat (KPK), Pakistan. *Int. J. Agric. Biol.* 15 857–863.

[B32] Ishida T. A. Nara K. Hogetsu T. (2007). Host effects on ectomycorrhizal fungal communities: insight from eight host species in mixed conifer-broadleaf forests. *New Phytol.* 174 430–440. 10.1111/j.1469-8137.2007.02016.x 17388905

[B33] James T. Y. Kauff F. Schoch C. L. Matheny P. B. Hofstetter V. Cox C. J. (2006). Reconstructing the early evolution of Fungi using a six-gene phylogeny. *Nature* 443 818–822. 10.1038/nature05110 17051209

[B34] Jany J.-L. Martin F. Garbaye J. (2003). Respiration activity of ectomycorrhizas from *Cenococcum geophilum* and *Lactarius* sp. in relation to soil water potential in five beech forests. *Plant Soil* 255 487–494. 10.1023/A:1026092714340

[B35] Johnson M. Zaretskaya I. Raytselis Y. Merezhuk Y. Mcginnis S. Madden T. L. (2008). NCBI BLAST: a better web interface. *Nucleic Acids Res.* 36 W5–W9. 10.1093/nar/gkn201 18440982 PMC2447716

[B36] Jones D. L. Hodge A. Kuzyakov Y. (2004). Plant and mycorrhizal regulation of rhizodeposition. *New Phytol.* 163 459–480. 10.1111/j.1469-8137.2004.01130.x 33873745

[B37] Kasel S. Bennett L. T. Tibbits J. (2008). Land use influences soil fungal community composition across central Victoria, south-eastern Australia. *Soil Biol. Biochem.* 40 1724–1732. 10.1016/j.soilbio.2008.02.011

[B38] Kirk P. M. Ainsworth G. C. Bisby G. R. International C. A. B. (2008). *Ainsworth & Bisby’s Dictionary of the Fungi.* Wallingford, CO: CABI.

[B39] Klarner B. Ehnes R. B. Erdmann G. Eitzinger B. Pollierer M. M. Maraun M. (2014). Trophic shift of soil animal species with forest type as indicated by stable isotope analysis. 123 1173–1181. 10.1111/j.1600-0706.2013.00939.x

[B40] Kõljalg U. Nilsson R. H. Abarenkov K. Tedersoo L. Taylor A. F. S. Bahram M. (2013). Towards a unified paradigm for sequence-based identification of fungi. *Mol. Ecol.* 22 5271–5277. 10.1111/mec.12481 24112409

[B41] Kutschera L. Lichtenegger E. (2002). *Wurzelatlas Mitteleuropäischer Waldbäume und Sträucher.* Graz: Stocker.

[B42] Lang C. Polle A. (2011). Ectomycorrhizal fungal diversity, tree diversity and root nutrient relations in a mixed xentral european forest. *Tree Physiol.* 31 531–538. 10.1093/treephys/tpr042 21636693

[B43] Lange M. Weisser W. W. Gossner M. M. Kowalski E. Türke M. Joner F. (2011). The impact of forest management on litter-dwelling invertebrates: a subtropical–temperate contrast. *Biodivers. Conserv.* 20 2133–2147. 10.1007/s10531-011-0078-0

[B44] Lauber C. L. Strickland M. S. Bradford M. A. Fierer N. (2008). The influence of soil properties on the structure of bacterial and fungal communities across land-use types. *Soil Biol. Biochem.* 40 2407–2415. 10.1016/j.soilbio.2008.05.021

[B45] Lazaruk L. W. Kernaghan G. Macdonald S. E. Khasa D. (2005). Effects of partial cutting on the ectomycorrhizae of Picea glaucaforests in northwestern Alberta. *Can. J. For. Res.* 35 1442–1454. 10.1139/x05-062

[B46] Li W. Godzik A. (2006). Cd-hit: a fast program for clustering and comparing large sets of protein or nucleotide sequences. *Bioinformatics* 22 1658–1659. 10.1093/bioinformatics/btl158 16731699

[B47] Lin W.-R. Chen W.-C. Wang P.-H. (2011). Effects of forest thinning on diversity and function of macrofungi and soil microbes. *Sydowia* 63 67–77.

[B48] Miyamoto Y. Sakai A. Hattori M. Nara K. (2015). Strong effect of climate on ectomycorrhizal fungal composition: evidence from range overlap between two mountains. *ISME J.* 9 1870–1879. 10.1038/ismej.2015.8 25647348 PMC4511943

[B49] Mölder A. Streit M. Schmidt W. (2014). When beech strikes back: how strict nature conservation reduces herb-layer diversity and productivity in Central European deciduous forests. *For. Ecol. Manage.* 319 51–61. 10.1016/j.foreco.2014.01.049

[B50] Nacke H. Thürmer A. Wollherr A. Will C. Hodac L. Herold N. (2011). Pyrosequencing-based assessment of bacterial community structure along different management types in german forest and grassland soils. *PLoS ONE* 6:e17000 10.1371/journal.pone.0017000 PMC3040199 21359220

[B51] Nehls U. (2008). Mastering ectomycorrhizal symbiosis: the impact of carbohydrates. *J. Exp. Bot.* 59 1097–1108. 10.1093/jxb/erm334 18272925

[B52] Oksanen J. Blanchet F. Kindt R. Legendre P. Minchin P. O’Hara R. (2015). *Vegan: Community Ecology Package. R Package version 2.0-0.* Oulu: University of Oulu. Available at: http://CRAN.R-project.org/package=vegan

[B53] Ott D. Digel C. Klarner B. Maraun M. Pollierer M. Rall B. C. (2014). Litter elemental stoichiometry and biomass densities of forest soil invertebrates. *Oikos* 123 1212–1223. 10.1111/oik.01670

[B54] Paillet Y. Berges L. Hjalten J. Odor P. Avon C. Bernhardt-Romermann M. (2010). Biodiversity differences between managed and unmanaged forests: meta-analysis of species richness in Europe. *Conserv. Biol.* 24 101–112. 10.1111/j.1523-1739.2009.01399.x 20121845

[B55] Pande V. Palm U. T. Singh S. (2004). Species diversity of ectomycorrhizal fungi associated with temperate forest of Western Himalaya: a preliminary. *Curr. Sci.* 86 1619–1623.

[B56] Peres-Neto P. R. Legendre P. Dray S. Borcard D. (2006). Variation partitioning of species data matrices: estimation and comparison of fractions. *Ecology* 87 2614–2625. 10.1890/0012-9658(2006)87[2614:VPOSDM]2.0.CO;2 17089669

[B57] Purahong W. Hoppe B. Kahl T. Schloter M. Schulze E. D. Bauhus J. (2014). Changes within a single land-use category alter microbial diversity and community structure: molecular evidence from wood-inhabiting fungi in forest ecosystems. *J. Environ. Manage.* 139 109–119. 10.1016/j.jenvman.2014.02.031 24681650

[B58] R Development Core Team (2008). *R: A Language and Environment Forstatistical Computing.* Vienna: Foundation for Statistical Computing.

[B59] Royo A. A. Carson W. P. (2006). On the formation of dense understory layers in forests worldwide: consequences and implications for forest dynamics, biodiversity, and succession. *Can. J. For. Res.* 36 1345–1362. 10.1890/08-1680.1

[B60] Schloss P. D. Westcott S. L. Ryabin T. Hall J. R. Hartmann M. Hollister E. B. (2009). Introducing mothur: open-source, platform-independent, community-supported software for describing and comparing microbial communities. *Appl. Environ. Microbiol.* 75 7537–7541. 10.1128/AEM.01541-09 19801464 PMC2786419

[B61] Shannon C. (1948). A mathematical theory of communication. *Bell Syst. Tech. J.* 27 379–423. 10.1002/j.1538-7305.1948.tb00917.x

[B62] Smith S. E. Read D. J. (2008). *Mycorrhizal Symbiosis.* London: Academic press.

[B63] Solly E. F. Schöning I. Boch S. Kandeler E. Marhan S. Michalzik B. (2014). Factors controlling decomposition rates of fine root litter in temperate forests and grasslands. *Plant Soil* 382 203–218. 10.1007/s11104-014-2151-4

[B64] Sterkenburg E. Bahr A. Brandstrom Durling M. Clemmensen K. E. Lindahl B. D. (2015). Changes in fungal communities along a boreal forest soil fertility gradient. *New Phytol.* 207 1145–1158. 10.1111/nph.13426 25952659

[B65] Suz L. M. Barsoum N. Benham S. Dietrich H. P. Fetzer K. D. Fischer R. (2014). Environmental drivers of ectomycorrhizal communities in Europe’s temperate oak forests. *Mol. Ecol.* 23 5628–5644. 10.1111/mec.12947 25277863

[B66] Tedersoo L. Bahram M. Põlme S. Kõljalg U. Yorou N. S. Wijesundera R. (2014). Global diversity and geography of soil fungi. *Science* 346:1256688 10.1126/science.1256688 25430773

[B67] Tedersoo L. May T. W. Smith M. E. (2010). Ectomycorrhizal lifestyle in fungi: global diversity, distribution, and evolution of phylogenetic lineages. *Mycorrhiza* 20 217–263. 10.1007/s00572-009-0274-x 20191371

[B68] Teste F. P. Lieffers V. J. Strelkov S. E. (2012). Ectomycorrhizal community responses to intensive forest management: thinning alters impacts of fertilization. *Plant Soil* 360 333–347. 10.1007/s11104-012-1231-6

[B69] Twieg B. D. Durall D. M. Simard S. W. (2007). Ectomycorrhizal fungal succession in mixed temperate forests. *New Phytol.* 176 437–447. 10.1111/j.1469-8137.2007.02173.x 17888121

[B70] Urbanová M. Šnajdr J. Baldrian P. (2015). Composition of fungal and bacterial communities in forest litter and soil is largely determined by dominant trees. *Soil Biol. Biochem.* 84 53–64. 10.1016/j.soilbio.2015.02.011

[B71] Vanbergen A. J. Woodcock B. A. Watt A. D. Niemelä J. (2005). Effect of land-use heterogeneity on carabid communities at the landscape scale. *Ecography* 28 3–16. 10.1111/j.0906-7590.2005.03991.x

[B72] Walker J. K. M. Phillips L. A. Jones M. D. (2014). Ectomycorrhizal fungal hyphae communities vary more along a pH and nitrogen gradient than between decayed wood and mineral soil microsites1. *Botany* 92 453–463. 10.1139/cjb-2013-0239

[B73] Wang X.-H. (2007). Type studies of *Lactarius* species published from China. *Mycologia* 99 253–268. 10.3852/mycologia.99.2.253 17682778

[B74] White T. Brans T. Lee S. Taylor J. (1990). “Amplification and direct sequencing of fungal ribosomal RNA genes for phylogenetics,” in *PCR Protocols: A Guide to Methods and Applications*, eds Innis M. A. Gelfand D. H. Sninsky J. White T. J. (San Diego, CA: Academic Press), 315–322.

[B75] Wickham H. (2009). *Ggplot2: Elegant Graphics for Data Analysis.* New York, NY: Springer.

[B76] Wingfield M. J. Slippers B. Roux J. Wingfield B. D. (2001). Worldwide movement of exotic forest fungi, especially in the tropics and the southern hemisphere this article examines the impact of fungal pathogens introduced in plantation forestry. *Bioscience* 51 134–140. 10.1641/0006-3568(2001)051[0134:WMOEFF]2.0.CO;2

[B77] Wubet T. Christ S. Schoning I. Boch S. Gawlich M. Schnabel B. (2012). Differences in soil fungal communities between European beech (*Fagus sylvatica* L.) dominated forests are related to soil and understory vegetation. *PLoS ONE* 7:e47500 10.1371/journal.pone.0047500 PMC3475711 23094057

[B78] Yurkov A. M. Kemler M. Begerow D. (2011). Species accumulation curves and incidence-based species richness estimators to appraise the diversity of cultivable yeasts from beech forest soils. *PLoS ONE* 6:e23671 10.1371/journal.pone.0023671 PMC3155558 21858201

