# Supplementary material for: Forest Management Type Influences Diversity and Community Composition of Soil Fungi across Temperate Forest Ecosystems
Source: Front Microbiol. 2015 Nov 24;6:1300. doi: 10.3389/fmicb.2015.01300 (PMC4656839; doi:10.3389/fmicb.2015.01300)
Supplement: Supplementary file 1 [file Data_Sheet_1.DOCX]

Supplementary Material

**Forest management type influences diversity and community composition of soil fungi across temperate forest ecosystems**

**Kezia Goldmann*, Ingo Schöning, François Buscot and Tesfaye Wubet**

*** Correspondence:** Kezia Goldmann, kezia.goldmann@ufz.de

# Supplementary Figures and Tables

## Supplementary Figures


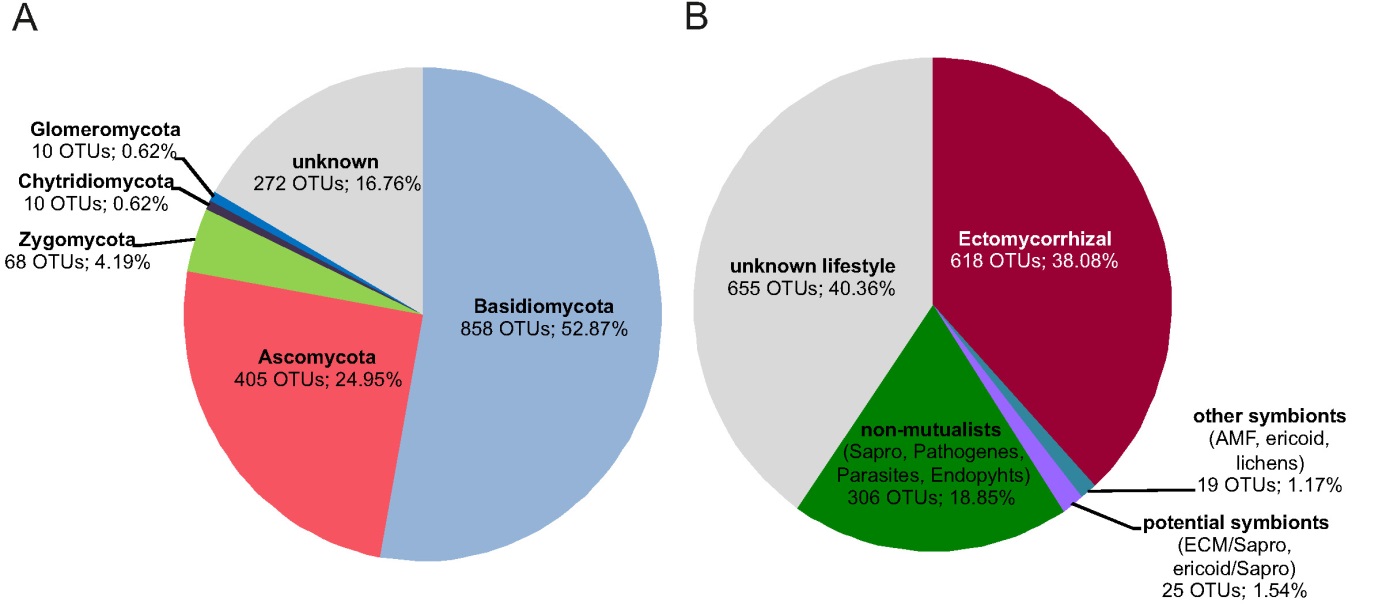


**Supplementary Figure S1.** Fungal OTU relative distribution: A–taxonomical classification at phylum level (name of phylum; number of OTU; percentage); B–classification of fungal ecological function at genus level (ecological function; number of OTU; percentage) based on literature research.


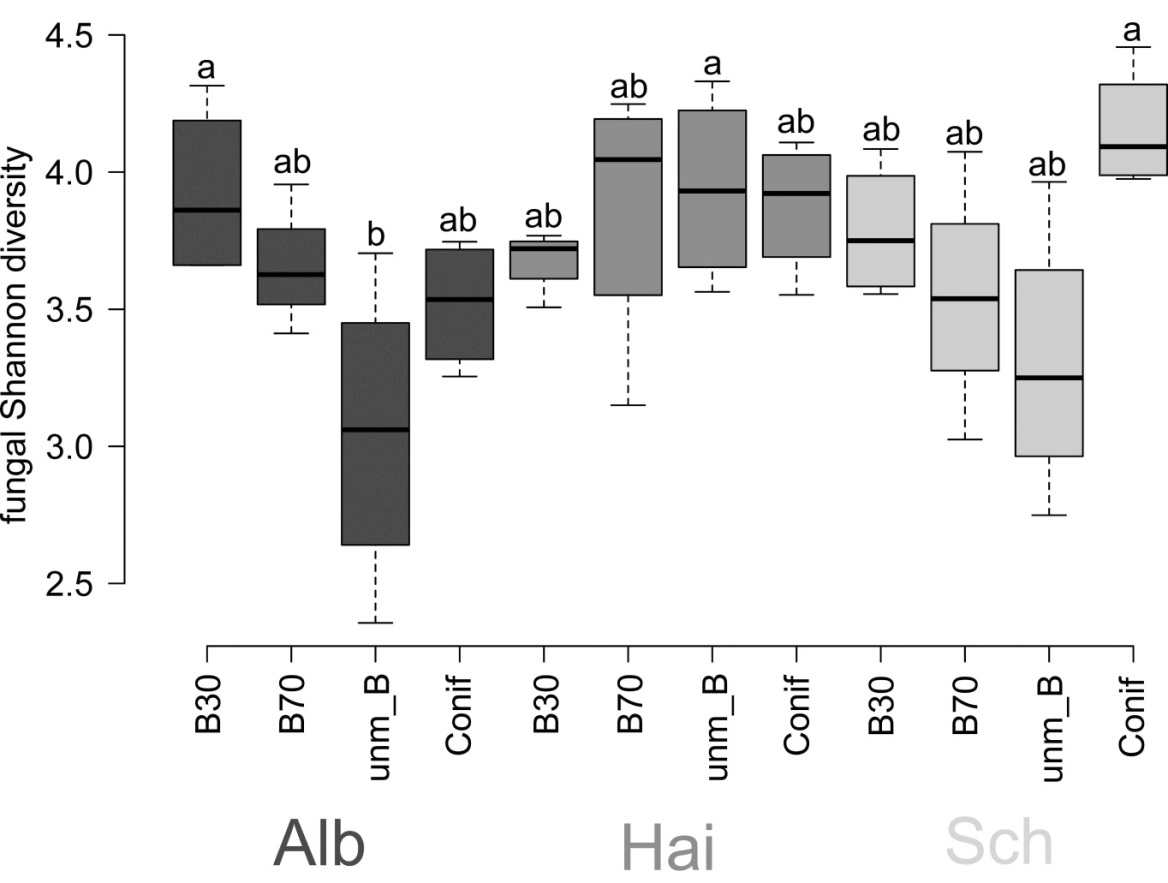


**Supplementary Figure S2.** Observed fungal Shannon diversity across the forest management types (young beech forest–B30, old beech forest–B70, coniferous forest–Conif, and unmanaged beech forest–unm_B) at the three study sites (Swabian Alb–Alb, Hainich-Dün–Hai and Schorfheide-Chorin–Sch. Different letters above bars indicate significant differences (p < 0.05) based on Tukey *post hoc* pairwise comparison.


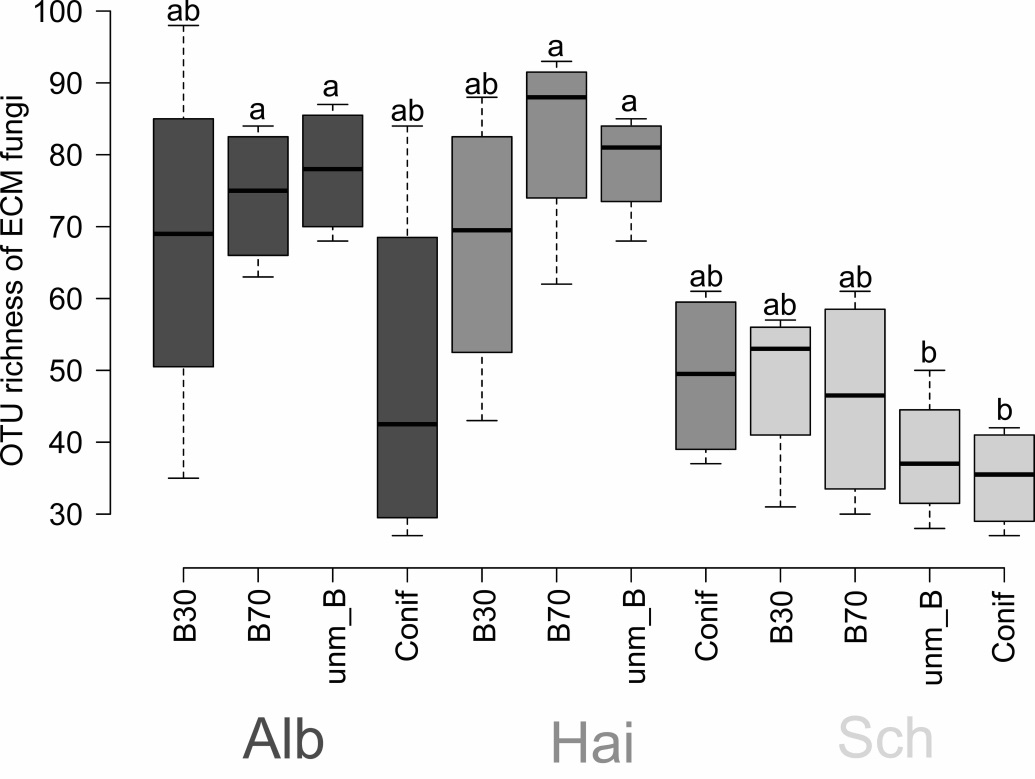


**Supplementary Figure S3.** Observed ectomycorrhizal fungal OTU richness across the forest management types (young beech forest–B30, old beech forest–B70, coniferous forest–Conif, and unmanaged beech forest–unm_B) at the three study sites (Swabian Alb–Alb, Hainich-Dün–Hai and Schorfheide-Chorin–Sch. Different letters above bars indicate significant differences (p < 0.05) based on Tukey *post hoc* pairwise comparison.





**Supplementary Figure S4.** The mean distribution of soil pH (top) and C/N ratio (bottom) across the three study sites Swabian Alb (Alb), Hainich-Dün (Hai) and Schorfheide-Chorin (Sch) and across the forest management types young beech forest (B30), old beech forest (B70), coniferous forest (Conif), and unmanaged beech forest (unm_B). Different letters above bars indicate significant differences between the forest managements types (p < 0.05) based on Tukey *post hoc* pairwise comparison.


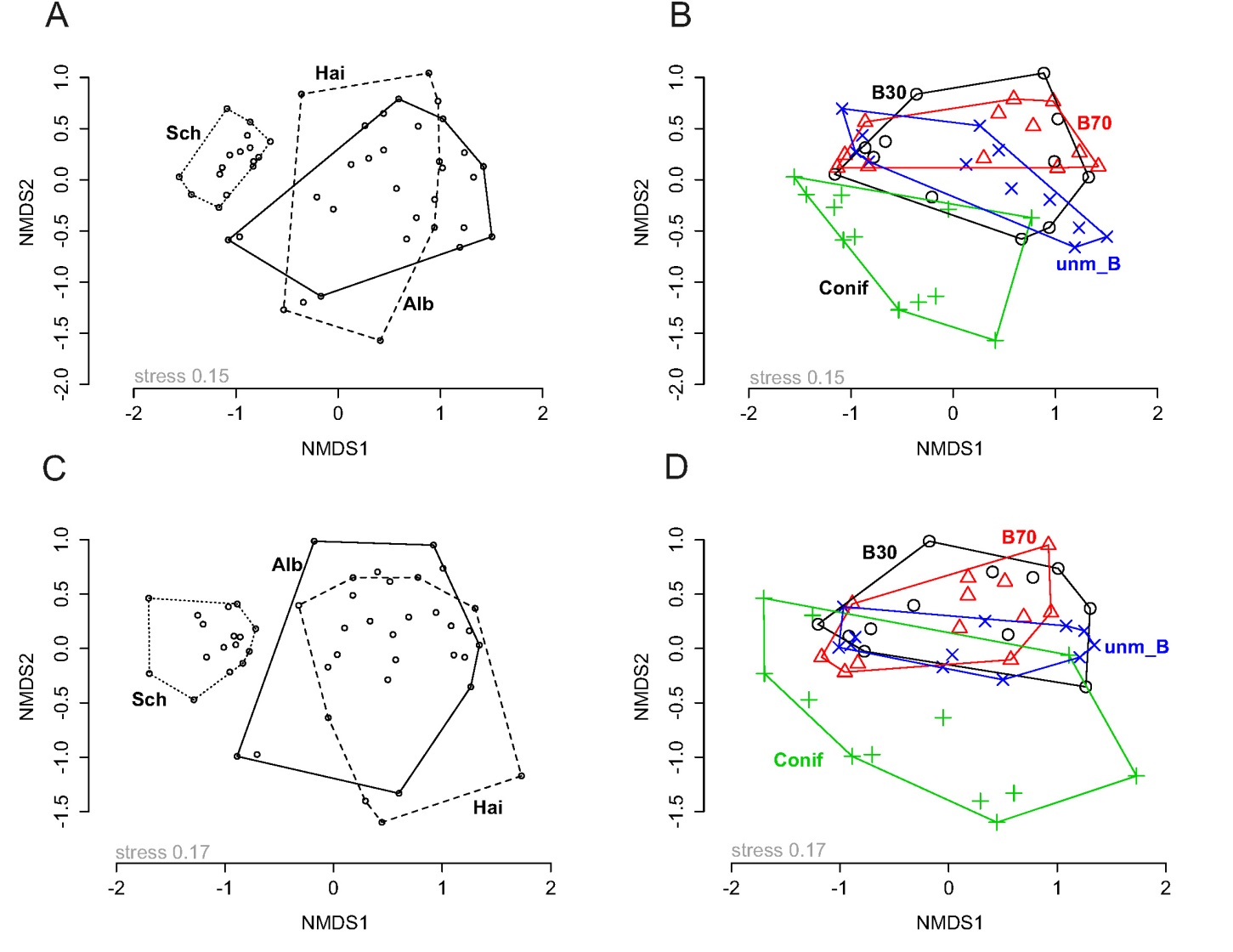


**Supplementary Figure S5.** NMDS ordination displaying the impact of: study site and forest management type on whole fungal community composition (A and B) and on ECM community composition (C and D). Abbreviations of study sites: Swabian Alb (Alb), Hainich-Dün (Hai) and Schorfheide-Chorin (Sch). Symbols and abbreviations for forest management type: young beech forest (о B30), old beech forest (∆ B70), coniferous forest (+ Conif), unmanaged beech forest(x unm_B). Stress values represent percentage.

## Supplementary Tables

**Supplementary Table S1.** Overview of the forest experimental plots examined, with the corresponding study site, forest management type and coordinates in each case. Abbreviations: young beech forest–B30, old beech forest–B70, coniferous forest–Conif and unmanaged beech forest–unm_B.

| **Plot** | **Study site** | **Forest management type** | **latitude** | **longitude** |
| --- | --- | --- | --- | --- |
| AEW01 | Swabian Alb | Conif | 48.47807317 | 9.334410162 |
| AEW02 | Swabian Alb | Conif | 48.37999048 | 9.351450642 |
| AEW03 | Swabian Alb | Conif | 48.4122625 | 9.355590784 |
| AEW04 | Swabian Alb | B30 | 48.39909865 | 9.244827343 |
| AEW05 | Swabian Alb | B70 | 48.41961847 | 9.414681796 |
| AEW06 | Swabian Alb | B70 | 48.39405132 | 9.445936708 |
| AEW07 | Swabian Alb | unm_B | 48.39623841 | 9.261355982 |
| AEW08 | Swabian Alb | unm_B | 48.38258955 | 9.382384493 |
| AEW09 | Swabian Alb | unm_B | 48.36934576 | 9.415217623 |
| AEW11 | Swabian Alb | Conif | 48.49159515 | 9.319752289 |
| AEW17 | Swabian Alb | B30 | 48.39645862 | 9.242991595 |
| AEW18 | Swabian Alb | B70 | 48.36500227 | 9.233799393 |
| AEW25 | Swabian Alb | B30 | 48.4758912 | 9.420956717 |
| AEW27 | Swabian Alb | B30 | 48.40023261 | 9.474351501 |
| AEW30 | Swabian Alb | B70 | 48.37273337 | 9.371377347 |
| AEW49 | Swabian Alb | unm_B | 48.44536875 | 9.476394288 |
| HEW01 | Hainich-Dün | Conif | 51.18535508 | 10.32362111 |
| HEW02 | Hainich-Dün | Conif | 51.21001129 | 10.36995394 |
| HEW03 | Hainich-Dün | Conif | 51.2716477 | 10.31073846 |
| HEW04 | Hainich-Dün | B30 | 51.36948156 | 10.53332754 |
| HEW05 | Hainich-Dün | B30 | 51.26387813 | 10.24095787 |
| HEW06 | Hainich-Dün | B70 | 51.26771983 | 10.23937826 |
| HEW10 | Hainich-Dün | unm_B | 51.08998735 | 10.46243387 |
| HEW11 | Hainich-Dün | unm_B | 51.10283444 | 10.40085432 |
| HEW12 | Hainich-Dün | unm_B | 51.1006881 | 10.45518298 |
| HEW13 | Hainich-Dün | Conif | 51.24264396 | 10.31294671 |
| HEW16 | Hainich-Dün | B30 | 51.18316036 | 10.36546587 |
| HEW17 | Hainich-Dün | B30 | 51.28281358 | 10.22690385 |
| HEW21 | Hainich-Dün | B70 | 51.1942783 | 10.31900538 |
| HEW22 | Hainich-Dün | B70 | 51.33715025 | 10.35927854 |
| HEW36 | Hainich-Dün | unm_B | 51.10627577 | 10.40930317 |
| HEW47 | Hainich-Dün | B70 | 51.17893474 | 10.37834387 |
| SEW01 | Schorfheide-Chorin | Conif | 52.90084729 | 13.84636695 |
| SEW02 | Schorfheide-Chorin | Conif | 52.95172886 | 13.77802812 |
| SEW03 | Schorfheide-Chorin | Conif | 52.92070744 | 13.6430016 |
| SEW04 | Schorfheide-Chorin | B30 | 52.91734663 | 13.84731137 |
| SEW05 | Schorfheide-Chorin | B70 | 53.05703356 | 13.88536623 |
| SEW06 | Schorfheide-Chorin | B30 | 52.90744279 | 13.84168805 |
| SEW07 | Schorfheide-Chorin | unm_B | 53.10734802 | 13.69441893 |
| SEW08 | Schorfheide-Chorin | unm_B | 53.19179716 | 13.93033786 |
| SEW09 | Schorfheide-Chorin | unm_B | 53.04458688 | 13.81010295 |
| SEW18 | Schorfheide-Chorin | Conif | 52.86242309 | 13.91724799 |
| SEW35 | Schorfheide-Chorin | B70 | 52.91126795 | 13.85341878 |
| SEW36 | Schorfheide-Chorin | B70 | 52.95136417 | 13.75414255 |
| SEW37 | Schorfheide-Chorin | B70 | 52.94002157 | 13.78261215 |
| SEW41 | Schorfheide-Chorin | B30 | 52.91229384 | 13.90992535 |
| SEW43 | Schorfheide-Chorin | B30 | 52.90097652 | 13.92832556 |
| SEW48 | Schorfheide-Chorin | unm_B | 53.05126592 | 13.8449954 |

**Supplementary Table S2.** Overview of number of sequences and fungal OTUs after different stages in the bioinformatic analysis: all sequences represent the whole dataset including rare OTUs, abundant sequences represent abundant OTUs (without singletons, doubletons and tripletons), re-normalized sequences represent the number of OTUs after a repeated normalization step after chimera removal.

|  | **Alb** | | **Hai** | | **Sch** | |
| --- | --- | --- | --- | --- | --- | --- |
|  | # Seqs | # OTU | # Seqs | # OTU | # Seqs | # OTU |
| all sequences | 31528 | 2453 | 31153 | 2635 | 32126 | 1658 |
| abundant sequences | 29703 | 1003 | 29173 | 1119 | 30806 | 667 |
| re-normalized sequences | 27392 | 993 | 27392 | 1113 | 27392 | 658 |

**Supplementary Table S3.** Correlation between general and ectomycorrhizal fungal OTU richness respectively Shannon diversity and soil chemical properties. p–significance value (in bold are p < 0.05), in brackets positve (+) or negative (-) correlations. Abbreviations: C_tot_–total carbon (sum of C_org_ and C_inorg_), C_inorg_–inorganic carbon, C_org_–organic carbon, N_tot_–total nitrogen, C/N ratio–ratio of organic carbon to total nitrogen.

|  | **C_tot_** | **C_inorg_** | **C_org_** | **N_tot_** | **C/N ratio** | **pH** |
| --- | --- | --- | --- | --- | --- | --- |
| OTU richness | 0.9365 | 0.3337 | 0.9085 | 0.8118 | 0.8913 | 0.201 |
| Shannon diversity | 0.5639 | 0.5665 | 0.543 | 0.7263 | 0.9995 | 0.3999 |
| ECM OTU richness | **<0.05 (+)** | 0.1163 | **<0.05 (+)** | **<0.05(+)** | **<0.001(-)** | **<0.05(+)** |
| ECM Shannon diversity | 0.7946 | 0.4806 | 0.81 | 0.317 | **<0.05(-)** | **<0.05(+)** |

**Supplementary Table S4.** Forest management types and abiotic soil characteristics shaping the fungal community composition at the three study sites, Swabian Alb (Alb), Hainich-Dün (Hai) and Schorfheide-Chorin (Sch), assessed by fitting environmental vectors (envfit, 999 permutations). r² - goodness of fit according to squared correlation coefficient, p–significance value (in bold are p < 0.05). Abbreviations: C_tot_–total carbon (sum of C_org_ and C_inorg_), C_inorg_–inorganic carbon, C_org_–organic carbon, N_tot_–total nitrogen, C/N ratio–ratio of organic carbon to total nitrogen.

|  | Alb | | Hai | | | Sch | | |
| --- | --- | --- | --- | --- | --- | --- | --- | --- |
|  | r² | *p* | | r² | *p* | | r² | *p* |
| Forest management type | 0.6261 | **<0.05** | | 0.5683 | **<0.05** | | 0.7031 | **<0.05** |
| C_tot_ | 0.0533 | 0.69 | | 0.5635 | **<0.05** | | 0.1354 | 0.373 |
| C_inorg_ | 0.142 | 0.38 | | 0.1321 | 0.432 | | 0 | NA |
| C_org_ | 0.0532 | 0.697 | | 0.5669 | **<0.05** | | 0.1354 | 0.373 |
| N_tot_ | 0.0176 | 0.892 | | 0.3902 | **<0.05** | | 0.0144 | 0.912 |
| C/N ratio | 0.7082 | **<0.05** | | 0.5584 | **<0.05** | | 0.3909 | **<0.05** |
| pH | 0.4609 | **<0.05** | | 0.4548 | **<0.05** | | 0.1222 | 0.42 |

**Supplementary Table S5.** Study site, forest management type and abiotic soil characteristics shaping the ECM fungal community composition. The r²–goodness of fit and p–significance value (in bold are p < 0.05) are calculated using the envfit function of vegan. Abbreviations: C_tot_–total carbon (sum of C_org_ and C_inorg_), C_inorg_–inorganic carbon, C_org_–organic carbon, N_tot_–total nitrogen, C/N-ratio–ration of carbon and nitrogen.

|  | **all sites** | | **Alb** | | **Hai** | | **Sch** | |
| --- | --- | --- | --- | --- | --- | --- | --- | --- |
|  | r² | *p* | r² | *p* | r² | *p* | r² | *p* |
| Study site | 0.5027 | **<0.05** | 0 | NA | 0 | NA | 0 | NA |
| Forest management type | 0.1607 | **<0.05** | 0.5901 | **<0.05** | 0.4677 | **<0.05** | 0.4974 | **<0.05** |
| C_tot_ | 0.5487 | **<0.05** | 0.0199 | 0.865 | 0.5925 | **<0.05** | 0.1441 | 0.398 |
| C_inorg_ | 0.2368 | **<0.05** | 0.151 | 0.365 | 0.5227 | **<0.05** | 0 | NA |
| C_org_ | 0.5444 | **<0.05** | 0.0166 | 0.887 | 0.5687 | **<0.05** | 0.1441 | 0.398 |
| N_tot_ | 0.6359 | **<0.05** | 0.0996 | 0.49 | 0.3896 | **<0.05** | 0.1729 | 0.303 |
| C/N ratio | 0.6478 | **<0.05** | 0.7135 | **<0.05** | 0.3703 | 0.062 | 0.3458 | 0.058 |
| pH | 0.7551 | **<0.05** | 0.5953 | **<0.05** | 0.7479 | **<0.05** | 0.1097 | 0.496 |

**Supplementary Table S6.** Correlation between A) C/N ratio, B) soil pH and most abundant ECM genera across the three study sites Swabian Alb (Alb), Hainich-Dün (Hai), Schorfheide-Chorin (Sch) and four forest management types young beech (B30), old beech (B70), unmanaged beech (unm B), coniferous (Conif) forest. Additional information regarding the exploration type (Agerer, 2001; Wei and Agerer, 2011; Shahin et al., 2013) and hydrophobicity (Lilleskov et al., 2011; Kühdorf et al., 2014) of each individual ECM genera are given. In bold p < 0.05; (+) positive or (-) negative correlations.

**A (C/N ratio)**

|  |  |  | Alb | | | | Hai | | | | Sch | | | |
| --- | --- | --- | --- | --- | --- | --- | --- | --- | --- | --- | --- | --- | --- | --- |
| Genera | Exploration type | Hydrophobicity | B30 | B70 | unm B | Conif | B30 | B70 | unm B | Conif | B30 | B70 | unm B | Conif |
| Russula | short/  medium-smooth | hydrophil | 0.8065 | 0.3805 | 0.7802 | 0.6922 | 0.7866 | 0.2930 | 0.8959 | 0.8890 | 0.2003 | 0.0794 | 0.4972 | 0.4010 |
| Inocybe | short | hydrophil | 0.6805 | 0.2599 | 0.8469 | 0.6098 | 0.4483 | 0.4145 | 0.6198 | 0.3689 | 0.9323 | 0.1923 | 0.7483 | 0.2721 |
| Sebacina | short/  medium-smooth | hydrophil | 0.4173 | 0.4838 | 0.0912 | 0.6783 | 0.3062 | 0.4922 | 0.447 | 0.9451 | NA | NA | NA | NA |
| Genea | short | no information | 0.3393 | 0.9046 | 0.5001 | NA | 0.4517 | 0.9338 | 0.1391 | 0.1770 | 0.3935 | 0.8622 | 0.3067 | **0.0243 (+)** |
| Lactarius | contact/short/ medium-smooth | hydrophil | 0.9358 | 0.7083 | 0.7939 | 0.4802 | 0.9352 | 0.8187 | 0.9324 | 0.3582 | 0.5301 | 0.1012 | 0.8904 | 0.0622 |
| Hygrophorus | contact/short | hydrophil | 0.1886 | 0.4333 | 0.2767 | 0.9998 | 0.1282 | 0.8932 | 0.5592 | 0.4351 | 0.1100 | NA | NA | 0.1489 |
| Elaphomyces | short | hydrophil | 0.1879 | 0.7818 | 0.3819 | 0.8977 | 0.4517 | 0.3856 | NA | 0.177 | 0.0612 | 0.1291 | 0.7642 | 0.5801 |
| Tomentella | contact/short/ medium-smooth | hydrophil | 0.466 | 0.0714 | 0.5253 | 0.3673 | 0.4719 | 0.8250 | 0.2133 | 0.2155 | 0.2717 | 0.8945 | 0.1432 | 0.5700 |
| Amanita | medium-smooth/long | hydrophob | 0.0967 | 0.3091 | 0.8751 | 0.9016 | 0.7731 | 0.1087 | 0.1462 | 0.7035 | 0.8467 | 0.7753 | 0.9058 | 0.5489 |
| Cortinarius | medium-fringe | hydrophob | 0.1634 | 0.3229 | 0.6633 | 0.8426 | 0.4868 | 0.2850 | 0.6763 | 0.1737 | 0.9521 | 0.6536 | 0.7785 | 0.7277 |
| Tylospora | short | hydrophil | 0.1879 | NA | 0.9458 | 0.7830 | NA | NA | NA | 0.6708 | 0.214 | NA | 0.0842 | 0.8034 |
| Hydnum | medium-fringe | hydrophob | 0.1879 | NA | NA | 0.9122 | NA | 0.087 | NA | NA | NA | NA | NA | NA |
| Piloderma | medium-fringe | hydrophob | 0.4206 | 0.6614 | **0.0455 (+)** | **0.0093 (-)** | 0.4379 | 0.1020 | 0.7513 | 0.8127 | 0.7122 | 0.5884 | 0.0542 | NA |
| Thelephora | medium-smooth | hydrophil | 0.1879 | 0.7966 | 0.2493 | 0.1937 | **0.0288 (+)** | 0.5545 | 0.3747 | 0.6241 | 0.9567 | 0.9964 | 0.8273 | 0.4227 |
| Boletus | long | hydrophob | 0.1879 | 0.9152 | 0.6574 | 0.6925 | 0.4646 | 0.1236 | 0.9203 | NA | 0.4383 | 0.9154 | 0.5126 | 0.7545 |
| Clavulina | contact/short | hydrophil? | 0.1879 | 0.778 | 0.4342 | 0.8815 | 0.6478 | 0.1084 | 0.0764 | 0.2624 | 0.8591 | NA | 0.9505 | NA |
| **B (pH)** |  |  |  |  |  |  |  |  |  |  |  |  |  |  |
| Russula | short/  medium-smooth | hydrophil | 0.6854 | 0.2201 | 0.4973 | 0.312 | 0.7971 | 0.1825 | 0.1942 | 0.4286 | 0.7983 | 0.2780 | 0.1316 | 0.3190 |
| Inocybe | short | hydrophil | 0.8363 | **0.0253 (+)** | 0.9060 | 0.9637 | 0.4271 | 0.4779 | 0.1211 | 0.4137 | 0.0915 | 0.6546 | 0.8691 | 0.8951 |
| Sebacina | short/ medium-smooth | hydrophil | 0.1148 | 0.9808 | 0.4336 | 0.4997 | 0.4541 | 0.1106 | 0.9059 | 0.0748 | NA | NA | NA | NA |
| Genea | short-distance | no information | **0.0449 (-)** | 0.7639 | 0.7534 | NA | 0.848 | 0.3439 | 0.8286 | 0.7125 | 0.5001 | 0.6275 | 0.1510 | 0.1210 |
| Lactarius | contact/short/ medium-smooth | hydrophil | 0.9614 | 0.2076 | 0.3278 | 0.3616 | 0.513 | 0.3169 | 0.5074 | 0.2471 | 0.5916 | 0.0948 | 0.3617 | 0.2595 |
| Hygrophorus | contact/short | hydrophil | 0.3425 | 0.9213 | 0.5859 | 0.4505 | **0.0368 (+)** | 0.6172 | 0.7172 | 0.1766 | 0.8917 | NA | NA | 0.0689 |
| Elaphomyces | short | hydrophil | 0.2165 | 0.8923 | 0.1207 | 0.532 | 0.848 | 0.3947 | NA | 0.7125 | 0.8438 | 0.4740 | 0.1534 | 0.9615 |
| Tomentella | contact/short/ medium-smooth | hydrophil | 0.8013 | 0.0794 | 0.7390 | 0.7414 | 0.8754 | 0.1782 | 0.7374 | 0.8082 | 0.6864 | 0.3710 | 0.7682 | 0.1275 |
| Amanita | medium-smooth/long | hydrophob | 0.2314 | 0.7461 | 0.8837 | 0.5286 | 0.8192 | 0.2035 | 0.9353 | 0.2358 | 0.7482 | 0.8489 | 0.6516 | 0.9647 |
| Cortinarius | medium-fringe | hydrophob | 0.1377 | 0.5685 | 0.7193 | 0.5821 | 0.8482 | 0.9065 | 0.2098 | 0.7172 | 0.432 | 0.9156 | 0.1399 | 0.8138 |
| Tylospora | short | hydrophil | 0.3158 | NA | 0.4459 | 0.8384 | NA | NA | NA | 0.2181 | 0.1617 | NA | 0.476 | 0.2716 |
| Hydnum | medium-fringe | hydrophob | 0.2165 | NA | NA | 0.5195 | NA | 0.2281 | NA | NA | NA | NA | NA | NA |
| Piloderma | medium-fringe | hydrophob | 0.9233 | 0.6691 | 0.1998 | 0.2355 | 0.2521 | 0.322 | 0.2532 | 0.1675 | 0.3129 | 0.7006 | 0.5559 | NA |
| Thelephora | medium-smooth | hydrophil | 0.2165 | 0.3984 | 0.5210 | 0.5902 | 0.1601 | 0.4168 | 0.7616 | 0.2533 | 0.1436 | 0.5447 | 0.4228 | 0.4131 |
| Boletus | long | hydrophob | 0.3158 | 0.6028 | 0.6050 | 0.7361 | 0.8654 | 0.7824 | 0.8971 | NA | 0.8575 | 0.5998 | 0.7768 | 0.2527 |
| Clavulina | contact/short | hydrophil? | 0.3158 | 0.4127 | 0.7993 | 0.7386 | 0.3359 | 0.7545 | 0.6156 | 0.7404 | 0.2644 | NA | 0.6587 | NA |

**References**

Agerer, R. (2001). Exploration types of ectomycorrhizae. *Mycorrhiza* 11**,** 107-114. doi: 10.1007/s005720100108.

Kühdorf, K., Münzenberger, B., Begerow, D., Karasch-Wittmann, C., Gómez-Laurito, J., and Hüttl, R.F. (2014). *Sebacina sp*. is a mycorrhizal partner of *Comarostaphylis arbutoides* (Ericaceae). *Mycol Prog* 13**,** 733-744. doi: 10.1007/s11557-013-0956-9.

Lilleskov, E.A., Hobbie, E.A., and Horton, T.R. (2011). Conservation of ectomycorrhizal fungi: exploring the linkages between functional and taxonomic responses to anthropogenic N deposition. *Fun Ecol* 4**,** 174-183. doi: 10.1016/j.funeco.2010.09.008.

Shahin, O., Paul, N.M.-S., Rambal, S., Joffre, R., and Richard, F. (2013). Ectomycorrhizal fungal diversity in *Quercus ilex* Mediterranean woodlands: variation among sites and over soil depth profiles in hyphal exploration types, species richness and community composition. *Symbiosis* 61**,** 1-12. doi: 10.1007/s13199-013-0252-0.

Wei, J., and Agerer, R. (2011). Two sebacinoid ectomycorrhizae on Chinese pine. *Mycorrhiza* 21**,** 105-115. doi: 10.1007/s00572-010-0312-8.
